# A Numerical Study on 3D Printed Cementitious Composites Mixes Subjected to Axial Compression

**DOI:** 10.3390/ma14226882

**Published:** 2021-11-15

**Authors:** Hanqiu Liu, King-James Idala Egbe, Haipeng Wang, Ali Matin Nazar, Pengcheng Jiao, Ronghua Zhu

**Affiliations:** 1 Institute of Marine Structures and Naval Architectures, Ocean College, Zhejiang University, Zhoushan 316021, China; liuhanqiu@zju.edu.cn; 2 Institute of Port, Coastal and Offshore Engineering, Ocean College, Zhejiang University, Zhoushan 316021, China; ekjames@zju.edu.cn (K.-J.I.E.); hpw@zju.edu.cn (H.W.); ali.matinnazar@zju.edu.cn (A.M.N.); 3 Engineering Research Center of Oceanic Sensing Technology and Equipment, Zhejiang University, Ministry of Education, Hangzhou 310000, China; 4 Yangjiang Offshore Wind Energy Laboratory, Yangjiang 529500, China

**Keywords:** numerical simulations, cementitious composites, 3D printed concrete, compression structural forms

## Abstract

Aptly enabled by recent developments in additive manufacturing technology, the concept of functionally grading some cementitious composites to improve structural compression forms is warranted. In this work, existing concrete models available in Abaqus Finite Element (FE) packages are utilized to simulate the performance of some cementitious composites numerically and apply them to functional grading using the multi-layer approach. If yielding good agreement with the experimental results, two-layer and three-layer models case combinations are developed to study the role of layer position and volume. The optimal and sub-optimal performance of the multi-layer concrete configurations based on compressive strength and sustained strains are assessed. The results of the models suggest that layer volume and position influence the performance of multi-layer concrete. It is observed that when there exists a substantial difference in material strengths between the concrete mixes that make up the various layers of a functionally graded structure, the influence of position and of material volume are significant in a two-layer configuration. In contrast, in a three-layer configuration, layer position is of minimal effect, and volume has a significant effect only if two of the three layers are made from the same material. Thus, a multilayered design approach to compression structures can significantly improve strength and strain performance. Finally, application scenarios on some structural compression forms are shown, and their future trajectory is discussed.

## 1. Introduction

The support for automation and complexities in design, in the architecture, engineering, and construction (AEC) industry, underpinned by a fast-paced concrete science and a materials engineering niche, are driving significant and rapid research in additive manufacturing (AM) [1] and constitutive material models [2,3,4] development and usage. As a result, applying additive manufacturing to concrete structures and numerical tools in modeling and predicting concrete behavior at the micro and macro scale is on the edge of new possibilities. Additive manufacturing (AM), otherwise referred to as three-dimensional concrete printing (3DCP), is an emerging method in the use of automation in construction [5]. The benefits of 3DCP include reduced construction time and cost, along with enhanced quality control and material efficiency [5]. Amidst the many benefits of 3DCP, the challenges of incompatibility between the layering process and reinforcement placement and the influence of process parameters remain [5,6]. 3DCP technology has been applied in a range of mixes, including eco-friendly mix ratios [7] and engineered cementitious composites (ECC) mixes [5]. More recently, mix designs have been developed to meet certain critical and time-dependent properties, such as flowability, buildability, and inter-layer adhesion [5]. In addition, eco-friendly mixes such as one-part geopolymer mix, rice husk ash, and innovative processes are increasingly being applied by researchers to improve buildability requirements optimize the performance of 3D printed concrete [8,9,10]. Over the last two decades, several materials mix designs incorporating various materials have been developed for various purposes and applied using various manufacturing techniques. These materials differ in scale, mechanical as well as chemical properties. Some concrete materials are developed to be eco-friendly, making use of environmental wastes and minimizing embodied carbon. Others are designed to optimize a set of objective functions, such as ECC, fiber reinforced concrete (FRC), or high-performance concrete (HPC), for optimized durability [11,12], compressive performance [11,13,14], tensile and flexural performance [15,16,17], crack mitigation [18], etc.

ECC, a special class of concrete mix, is renowned for its tensile strain-hardening behavior and tensile ductility because constituent materials like fiber, fly ash, and microsilica [5] are being used as print materials in 3DCP. Fiber addition has been reported to weaken inter-layer bond strength, induce stress concentration, and reduce performance [19]. However, by adjusting the nozzle diameter, fibers can be re-aligned, and improvements have been reported in the flexural performance of 3D printed fiber reinforced cement paste when the fibers are aligned [20,21]. Microsilica, also known as microsilica, is a widely used constituent of ECCs. The addition of microsilica up to 10% volume of cement in concrete mix designs increases the mix viscosity and short term [22,23] and long-term compressive strength [24]. However, at high volumes above 15%, it reduces packing density and ultimately produces a decline in the compressive strength [25]. The use of microsilica also significantly increased the flexural performance of concrete [26], a trend that increases with increased microsilica content. In 3D printed foam concrete, microsilica increased the static yield stress significantly [27]. Therefore, the addition of microsilica to concrete as a replacement for Ordinary Portland Cement (OPC) has its merits within a range. The effects of microsilica as a replacement for fine aggregates in 3D printed concrete have not been considered.

A performance-driven approach (PDA) is required in structural design and infrastructure construction to reap the benefits of the multitude of materials studied and developed over time. PDA is simple, conforming structural design and material used to a set of the performance function. While the concept of PDA is not new, the reluctance to apply it in real projects lingers [28]. Often this is the case because most material mix designs are not standardized as inputs for numerical design software and structural design codes. Another reason is that the performance of the resulting structural component or structural system depends on the individual properties of the constituting materials and their interaction with other fillers and cementitious composites. Thus, structural engineers may prefer to play safe and utilize materials familiar to them and easy to implement in the field than using a relatively complex material mix or method irrespective of its benefits. PDA may take different approaches to concrete composites; an example is a special monolithic mix for a given structural component, such as columns or beams. It may also take the functional grading approach within the overall matrix of a structural unit or system such as road, bridge pier, deck, or shells.

The urgent need to cut down global carbon emissions coupled with advances in AEC has led to a significant rise in interest in functionally graded concrete (FGC) [29,30]. FGC, often realistically applied as multi-layer concrete, entails varying the mechanical properties of a structural element spatially within a region by altering the material composition across that space according to some specific design function [30,31]. As is observed, FGC converges with PDA as both concepts attempt to optimize sets of performance objectives. Therefore, it can be speculated that FGC is a subset of PDA, and the application of FGC using PDA will minimize cost while maximizing efficiency and performance. In practice, FGC is implemented using a fresh-on-fresh approach or fresh-on-hardened approach [30]; this study closely relates to the fresh-on-hardened approach. Apart from material properties, a variety of factors may influence the performance of FGC, such as position, relative thickness, and interlayer stiffness. The fracture behavior of FGC materials for rigid pavement was influenced by the position of the FRC layer as assessed in [32]. Yager et al. [29] also showed that using FRC layers in varying thicknesses of 20, 35, and 50 mm) at bottom positions in FGC beams improved the cracking load but not the load capacity. However, Sridhar and Prasad [33] reported static load increase with increasing hybridized FRC layer of 25, 50, and 75 mm, respectively. FGC has been applied to assess the potential for use with recycled aggregates [34,35] and mitigate durability issues in wind turbine towers [36,37]. It has also been used in assessing and applying waste rubber tires in construction [38,39]. Recently, an automated system has been developed to 3D print FGC-based materials [1], creating more avenues for new FGC combinations and achieving a smooth gradation.

The mechanical performance of a new concrete structure or mix is primarily studied by experimentation. These tests make it possible to evaluate the structural performance of materials in basic geometries of cubes, cylinders, and rectangular beams. Considering the current AM technology, the compressive performance of 3D printed concrete cubes is expected to be lower than mold cast samples because of the inadequacies of the filament layer interface, zero compaction approach, and unavoidable voids that develop during the printing process [5,6,40]. More so, the strength will vary with printed layer orientation and loading direction for un-cored samples [6,21]. However, Rahul et al. [40] reported minimal variation in compressive strength for cored samples from 3D printed walls. Evaluating concrete by experimentation requires the investment of time and financial resources that are often limited, especially when full-scale parametric studies or performance predictions are required to assess the behavior of a material under changing conditions, as is the case in multi-layer concrete or FGC. As a result, validated numerical models are often used to simulate material performance to relatively high accuracy, considering loading and boundary conditions that mimic real-life situations. FE modeling has been implemented to study a range of physical problems, such as reinforced concrete beams [41] and beams subjected to shear [42]. FE tools have also been applied in modeling and assessing FGC components for fracture resistance [43] and in developing a blueprint for such components [44]. Thus, using FE tools, the performance and application of FGC to structural compression forms can also be assessed.

Existing FE tools, such as Abaqus CAE, can utilize experimental data from concrete testing, converted into model inputs, enabling design, analysis, and numerical performance evaluation and prediction. Many studies have been carried out on the flexural performance of FGCs. However, very few studies consider the application of FGC in compression forms, and none exists on the compressive performance of multilayered cementitious composites. In this work, glass fiber and microsilica dominant cementitious composites are developed and test their performance tested. These materials are modeled in Abaqus, and their results are compared to experimental performance. The validated material model is applied to numerical studies on FGC in compression by the multi-layer approach. The rest of the paper is presented as follows: Section 2 presents the materials and method applied in the study, summarizing the principles of three material models, out of which two are applied in this work. Section 3 discusses the results and Section 4 conceptualizes the applications of FGC in structural compression forms. Section 5 summarizes the main findings.

## 2. Materials and Method

### 2.1. Fabrication and Testing

To manufacture and assess the mechanical performance of 3D concrete samples, the concrete mixes’ pumping and shape retention requirements had to be satisfied, ensuring minimal geometric deviation of hardened samples. Therefore, the mixes’ shape retention index (SRI) is determined as the ratio between the bottom width of an extruded paste filament and the printer nozzle’s width. The ingredients used include: fine granite aggregates, with particles up to 98% passing 2 mm sieve and a specific gravity of 1.95. GF, OPC, Grade 42.5, microsilica, rapid hardening cement with a specific gravity of 3.1, water reducing agent (polycarboxylic acid series) with a water reduction rate of 20–25%, and expansion agent: alkali content 0.4% fineness 10% powder were also used. The chemical composition of the microsilica used in the mix is shown in Table 1, while the properties of the glass fiber are presented in Table 2. Three classes of mixes were made, the control, microsilica concrete (MC), and glass fiber concrete (GFC) mix leading to a total of seven unique mix compositions as presented in Table 3. The water to cementitious materials ratio was 0.35 for all mix compositions. In MC, the fine aggregates were replaced by microsilica, and in GF mixes, glass fiber was introduced in varying volumes.

**Figure 1:**
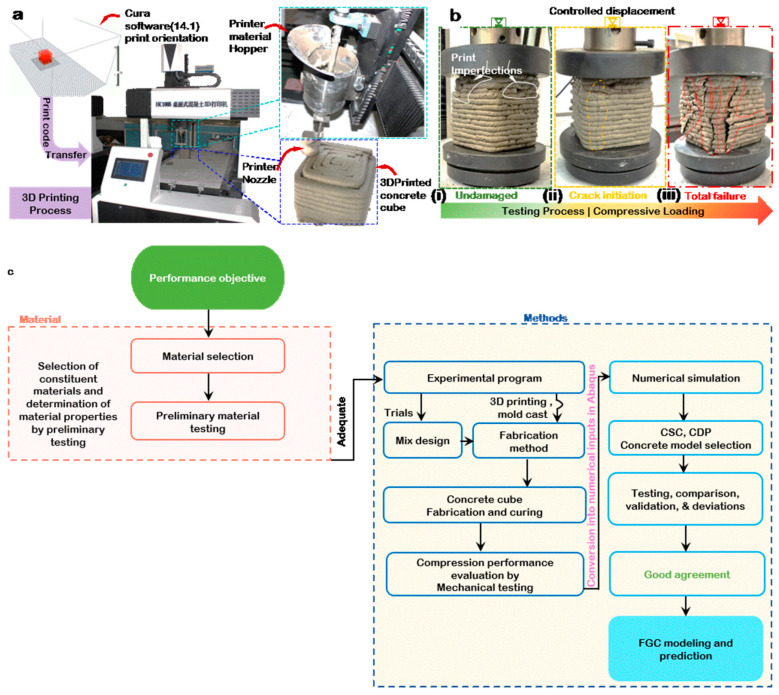
(**a**) 3D concrete model printing process. (**b**) Compressive strength test showing loading and failure. (**c**) Workflow of the program.

The 3D modeled cube is transferred to the 3D printer software (Cura 14.1, CIIC Technology Co., Ltd., Tianjin, China) interface and oriented to an appropriate print direction, as shown in Figure 1a. The solid ingredients were initially mixed dry for 3–5 min to ensure a homogenous mix of the concrete constituent and proper fiber dispersion; subsequently, the liquid ingredients and water were introduced. A constant amount of 100 kg/m^3^ of rapid hardening cement, 0.1% vol. water reducing agent, and 0.35% vol. sulphoaluminate-based expansion agent is utilized in the mix to compensate for the shrinkage caused in concrete due to hydration, and 270 kg/m^3^ of water was used. After sufficient consistency is attained, the mix is introduced into the 3D printer’s material hopper, i.e., uniform rheology that satisfies good 3D printing buildability thorough mixing of the concrete mix materials to attain a suitable viscous blend that minimizes bulging or collapse in its fresh state and geometric deviation of hardened samples.

Then, the printing is executed using a robotic arm, extrusion type concrete 3D printer by CIIC, Model HC1008 (CIIC Technology Co., Ltd., Tianjin, China), as illustrated in Figure 1a. Care was taken to ensure consistency in printing to guarantee repeatability within the margins of allowable experimental errors. Printing parameters can be defined from the 3D printer directly. The nozzle diameter was 15 mm, and the dimension of each extruded filament was about 15 mm × 7.2 mm, width by height, respectively. The nozzle speed and extrusion velocity were 20 mm/s. The cube dimension was 100 mm × 100 mm × 100 mm, length, width, and height, respectively.

**Figure 2:**
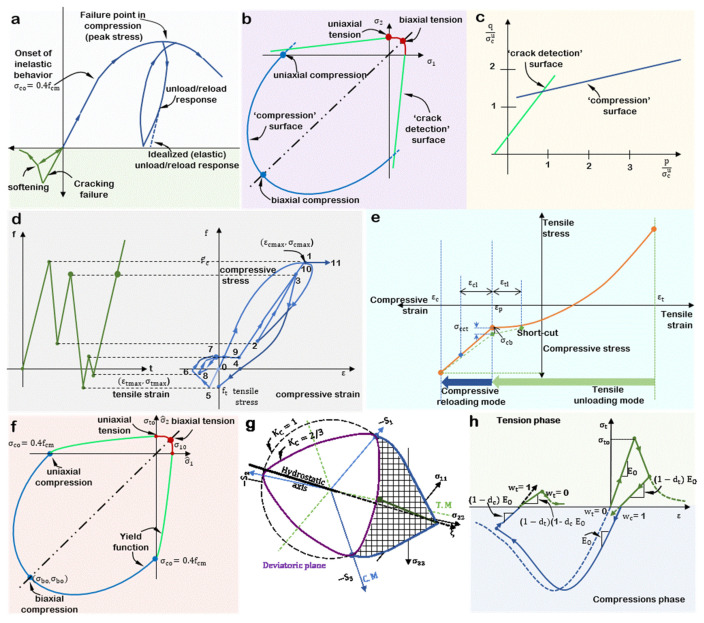
Some concrete models available in commercial FE numerical software. (**a**) Uniaxial behavior of plain concrete. (**b**) Yield and failure surface in plane stress for CSC model in Abaqus. (**c**) Yield and failure surface in the (p–q) plane for CSC model in Abaqus. (**d**) Hysteresis for Maekawa model from Diana FEA. (**e**) Crack-reclosing behavior for Maekawa model from Diana FEA. (**f**) Yield surface in plane stress for CDP model in Abaqus. (**g**) Yield surfaces in the deviatoric plane for the CDP model in Abaqus. (**h**) Uniaxial load cycle for CDP model in Abaqus.

After printing, a normal curing method was applied. The specimens were left to harden for 24 h at room temperature while covered with plastic sheets to minimize evaporation. Afterward, the specimen was cured in a water bath for 28 days at about 28 °C. A compressive strength test on the cube samples was carried out by loading them perpendicular to the print direction, the Z-direction, as shown in Figure 2b. In accordance with BS EN 12390-3:2019 [45]. The load is controlled by displacement at 1 mm/min, and the testing machine is controlled by a microcomputer electronic universal testing machine (UTM model WD-P 6305, Minghu Testing Instrument Co., Ltd., Jinan, China) to determine all stress and strain data. Care was taken to follow standard testing procedures and guarantee approximate repeatability. Figure 1b(i) shows the undamaged sample with print imperfections. The effects of these imperfections on overall material performance are not studied in this work. However, it should be noted that, in some cases, and depending on the severity, 3D printing imperfections can significantly reduce the ultimate performance of concrete mixes [6,7]. Figure 1b(ii) shows the onset of macro cracks and visible failure. As loading continues, the cracks widen, and more cracks develop and coalesce, leading to total failure, as shown in Figure 1a(iii). The workflow of the study is summarized in Figure 1c.

### 2.2. Performance Evaluation and Prediction by Modeling

Concrete can be modeled in commercial FE software using applied damage theories. The ability to transform experimental data into numerical material models has improved studying complex isotropic materials like concrete. Concrete damage models (CDM) can simulate concrete degradation resulting from the microstructural coalescence of micro-cracks under applied load. The behavior of the degrading concrete under load can be evaluated using failure theories such as the continuum damage mechanics theory (CDMT). CDMT is concerned with the representation of the failure of materials, modeled as a continuous mass rather than as discrete particles to illustrate the initiation, propagation, and fracture of solid materials without resorting to microscopic constitutive details that are complex for practical engineering purposes such as analysis and predictions [46,47]. Often, practical CDMs utilize yield surfaces to represent a failure theory in the principal stress space. Based on the principles of continuum mechanics, CDM is governed by sets of defined variables that modify the stiffness matrices of a concrete model following predefined elasticity damage or plasticity theories or both. Another essential failure theory used in the modeling of concrete is the Modified-Compression Field Theory (MCFT). Originally proposed by Vecchio and Collins in 1986 [48], MCFT was developed for modeling reinforced concrete response in the 2D space. MCFT is a model for the load-deformation response of two-dimensional cracked reinforced concrete subjected to shear. It models concrete behavior based on concrete stresses in principal directions summed with reinforcing stresses assumed to be purely axial. It was later expanded in 1995 by Selby and Vecchio [49] to cover general three-dimensional analysis of reinforced concrete solids.

#### 2.2.1. Abaqus Concrete Smeared Cracking (CSC)

One of the popular CDMs, based on CDMT, is concrete smeared cracking (CSC). Smeared crack models adopt cracking as distributed effect with directionality. The cracked material is simulated as a continuous medium with anisotropic properties arising with each crack opening at each integration point [50]. Various concrete models and finite element programs adopt the CSC in their approach. For example, the CSC model in Abaqus, developed for monotonic loading conditions, is cracking-based. Essentially, cracking behavior and its detection are the baseline of the modeling approach. The uniaxial response of the incorporated model in the compression and tension phases is shown in Figure 2a.

When concrete is loaded in compression beyond its elastic regime in the CSC model, it develops inelastic non-recoverable strains. If it is unloaded before the failure point, the reloaded elastic response will be softer than the initial. However, the model assumes that the post-failure response in tension is governed by damaged elasticity, not unrecoverable or permanent straining (plasticity), thus allowing cracks to close and open when the loading changes from a tensile to a compressive state and vice versa. Tensile degradation is determined by a crack detection surface, as shown in Figure 2b, and taken as the coulomb line, as shown in Figure 2c, a function of the first two stress invariants. See [50] for more details. Failure is modeled by elastic-plastic theory using a yield surface, isotropic hardening, and associated flow in a dominantly compressive state. The compressive yield surface is also written in terms of its first two stress invariants. Designed for monotonic loading cases, the model is not particularly developed to include the prediction of the cyclic response of concrete or the reduction in elastic stiffness resulting from inelastic straining. However, results may not be far off if applied to such cases.

#### 2.2.2. Maekawa–Fukuura Concrete Model (MFC)

Another CSC-based concrete model is the MFC model available in Diana FEA V10.5. It is a combination of three concrete models: the Total Strain crack model (TSM), the elastoplastic damage model (EPM), and the Maekawa Cracked concrete (MCC) curves. TSM is based on CSC and developed along the lines of MCFT. The basic building block is hypo-elasticity, where the loading and unloading behavior is along the same stress–strain path as shown in Figure 2d. The cracks are always orthogonal, either fixed with the coordinate system or rotating with the principal directions of the strain vector. Both approaches are possible within the same TSM framework. However, the Maekawa–Fukuura concrete model uses a non-orthogonal crack definition [51]. In other words, a user-defined minimum threshold angle can be specified between any two cracks in the same integration point. An angle lower than 90°, e.g., 45° or 22.5°, permits more than three cracks to form, and up to a maximum of six per integration point but at different times. In general, the underlying concept of the TSM is that the stresses are evaluated in the directions resulting from the crack orientation. The strain vector $\left(\varepsilon_{xyz}\right)$ in the element coordinate system xyz is updated with the strain increment $\left(∆\varepsilon_{xyz}\right)$ according to

(1) εi+1t+∆txyz=εtxyz εi+1t+∆txyz

The EPM is based on a fracture parameter denoted as “K,” which ranges from 0 (complete damage) to 1 (intact state). K reduces the shear modulus of the concrete. Including defects, K represents the degradation of the shear elastic strain energy of the concrete. Once the elastic strain vector denoted as “F” is determined at any integration point, K is calculated as a function of the invariants of the elastic strain tensor and some elastic variables. The calculated factor reduces the shear modulus at that point, and the model moves to the next integration step. However, to calculate the resulting damage shear modulus, the precise stresses and the elastic-moduli matrix of the damaged concrete are formulated primarily as a function of the damage parameter (of the invariants of the elastic strain tensor). Plastic hardening denoted by “H” is defined by the second strain invariant and a user-defined correction factor for plastic evolution. Plastic dilatancy, also known as plastic flow, results from shear plastic dislocation along the lines of the internal defect, and it is a function of the first strain invariant and K. When MFC is selected in Diana FEA for compression models, EPM is applied until the first crack occurs in an integration point. When cracking occurs at an integration point, the model changes to a non-orthogonal Total Strain crack model. The parameters F, K, and the compressive and tensile stresses and strains at the integration point, being the controlling parameters for both models, are transferred [51].

The MCC curves are shown in Figure 2d, which are uniaxial stress–strain curves for loading, unloading, and reloading conditions in the tension and compression strain domains: 0–1, Compressive loading; 1–2, Compressive unloading; Compressive reloading; 3–4, Compressive unloading; 4–5, Tensile loading; 5–6, Tensile loading; 6–7, Tensile unloading; 7–8, Tensile reloading; 8–9, Tensile unloading; 9–10, Compressive reloading; 10–11, Compressive loading. Each zone of the MCC is defined by empirical relation between a range of the parameter, including but not limited to K, stress, strain, and the young’s modulus of the concrete. The crack reclosing option can be defined as it relates to the compressive reloading and the tensile unloading behavior of the model, as shown in Figure 2e. Tension softening can be defined from a selection of available softening curves available in the model.

#### 2.2.3. Concrete Damaged Plasticity (CDP)

The CDP model, available in Abaqus CAE, is multi-hardening plasticity and isotropic damaged elasticity-based model [52]. The Drucker–Prager strength hypothesis is the basis for the yield function and non-linear plasticity governing equation [52,53]. First put forward by Lubliner in 1989 [54], the compression and tensile yield surfaces are governed by the Drucker–Prager criterion, with a non-associative plastic flow controlled by the Drucker–Prager hyperbolic flow potential. See [53,55]. Stiffness loss and bi-isotropic hardening were later introduced to the model by [53] to capture the evolution of strength in tension and compression as shown in the yield surface in Figure 2f. The yield function is defined primarily in terms of the von mises equivalent stress, the hydrostatic pressure stress, and the maximum principal effective stress amidst other dimensionless ratios. More details can be found here [52,53,54,55,56,57,58]. Figure 2g shows the yield surface in the deviatoric plane governed by $K_{c}$, which is the ratio of the second stress invariant on the tensile meridian, i.e., (the distance between the hydrostatic axis and the tensile meridian) to that on the compressive median, i.e., (the distance between the hydrostatic axis and the compressive meridian) at initial yield. $K_{c}$ varies between 0.5 and 1, where it becomes a circle as reported in the original Drucker–Prager strength hypothesis. [53]. The default value of $K_{c}$, in Abaqus is 2/3.

The CDP assumes the failure mechanisms for failure are tensile cracking and compressive crushing. Failure is regulated by plastic strain in tension $\varepsilon_{t}^{\sim pl}$ and compression $\varepsilon_{c}^{\sim pl}$, the two hardening variables that ensure the initiation of inelastic behavior at approximately $0.4f_{cm}$ and $0.4f_{tm}$ under uniaxial and biaxial compression and tension. A vital aspect of this model is its ability to model cyclic behavior because of the “unilateral effect”, the behavior of the models’ elastic stiffness recovery as tensile cracks close when loading changes from tension to compression, as shown in Figure 2h. The gradual loss in elastic modulus (E) is governed by a scalar elastic degradation variable “*d*” as

(2) $$E=1\left(1-d\right)E_{o}$$

where $E_{o}$ is the undamaged modulus of the model. “*d*” varies from zero to one, with zero corresponding to an intact state and one corresponding to the total failure of the model. The expression is valid in the tension and compression phases of the cycle. The variable connoting degradation “*d*” is a function of the stress and uniaxial damage variables $d_{t}$ (tensile damage parameter) and $d_{c}$ (compression damage parameter).

A comparison of the models is presented in Table 4, and the essentials and implementation of the CDP model are presented in the Appendix A.

## 3. Results and Discussion

### 3.1. Compressive Performance

**Figure 3:**
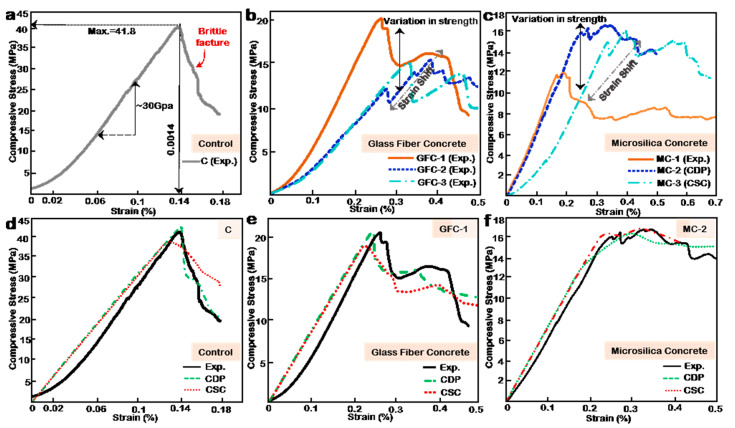
(**a**) Compressive stress–strain curve for the control sample. (**b**) Compressive stress–strain curve for microsilica concrete samples. (**c**) Compressive stress–strain curve for glass fiber concrete samples. (**d**) Numerical simulation and validation of the control sample using Abaqus CSC and CDP models. (**e**) Numerical simulation and validation of GFC-1 sample using Abaqus CSC and CDP models. (**f**) Numerical simulation and validation of MC-2 sample using Abaqus CSC and CDP models.

Figure 3a presents the experimental result for the compression test of the normal concrete control sample. Perhaps, because of the absence of coarse aggregates, the compression performance of the sample showed minimal hardening, i.e., normal concrete incorporating coarse aggregates show a more visible hardening phase in transition from the elastic to inelastic regime due to their higher resistance to creep. Furthermore, it exhibited rapid softening and an exponential post-failure curve. In Figure 3b, the results from the GFC samples are presented. A multi-peak stress–strain relation owing to the bridging effect provided by the glass fibers in the concrete matrix is observed. The intermittent stiffening and increase in ultimate strain are a vital improvement in the brittle nature of concrete. However, in 3D printed concrete, glass fibers can reduce interlayer bond strength [19], increase print imperfections and micropores, ultimately reducing compressive strength. The observed decrease in compressive strength with an increase in fiber volume is thus attributed to print imperfections and micropores. These demerits mentioned above do not exist in traditionally fabricated samples. Figure 3c shows the stress–strain curve for MC samples. Although multiple peaks are observed in MC samples, the most notable trend is the extended strains. The microsilica replacement mix was best at 12% volume fine aggregate replacement (MC-2). In comparison to C and GFC samples, MC samples can sustain large deformation without significant damage. Nevertheless, their ultimate compressive strengths are significantly less than C and fairly lower GFC. MC mixes show that, at a high volume of microsilica, the brittle nature associated concrete can be reduced for normal-strength concrete. The extended strain may result from the combined effect of glass fibers and microsilica as microsilica has been shown to improve the compressive performance of concrete, and its synergy with fiber has also shown a more significant effect. Although, the MC- samples contain a significant amount of microsilica and a minimal fiber volume, the observed behavior may be accrued more to microsilica than glass fiber [21,22,23].

### 3.2. Numerical Modeling of the Cubes, Result Comparison and Validation

The numerical models are only developed using the CSC and CDP modeling theories in the Abaqus Computer-Aided Engineering, FE software. The simulation and experimental results are compared to validate the performance of both models. See the Appendix A for the models’ modeling procedure, material input data, and yield and stress contour plots. Figure 3d compares the performance of the models using the control sample. It is observed that the CDP model outperforms the CSC model. Furthermore, the accuracy of the CDP model in both the pre-and post-failure regime is high, achieving excellent agreement with the experimental plot.

In contrast, the CSC model predicts the elastic performance to a good degree but fails to capture the post-failure softening adequately. In general, the performance of both models is satisfactory. In Figure 3e, both models capture the general trend of GFC-2, but their inelastic stress–strain performance is not significantly accurate as observed in the control sample. The resulting average performance in the inelastic range may be attributed to insufficient input data in the tensile regime of the models and the multi-peak nature of the stress–strain curve. Figure 3f shows excellent agreement between the experimental result, the CSC, and CDP models. Both models accurately mimic the performance of the sample in both the elastic and inelastic regimes. It is seen that both models are sufficiently accurate and can be applied to study other structural forms numerically. Based on enhanced accuracy and the widespread usage of the CDP model, it is applied to study FGC by the multilayering approach.

### 3.3. Modulation of FGC by Multilayering Approach Using CDP

**Figure 4:**
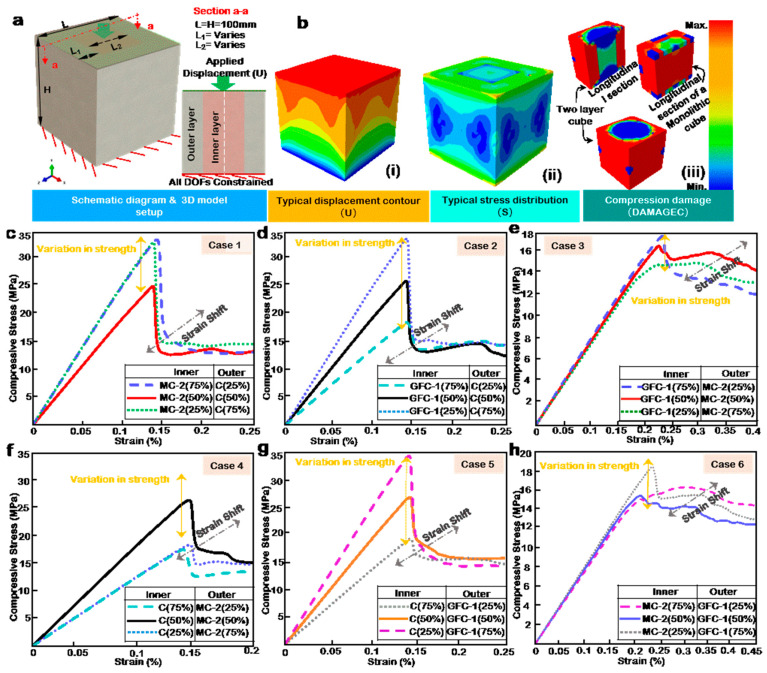
(**a**) Schematic diagram of the two-layer configuration, showing the applied displacement and boundary condition. (**b**) Two-layer configuration simulation contour plot of (**i**) Displacement, (**ii**) Misses stress, and (**iii**) Compression damage. Numerical stress–strain curve for (**c**) Case 1, (**d**) Case 2, (**e**) Case 3, (**f**) Case 4, (**g**) Case 5, and (**h**) Case 6.

Multilayering in this case and study refers to a heterogeneous array of concrete mixes within a given structural form and should not be mistaken for layered 3DCP. For a cost-efficient and high-performance concrete structure, multilayering may often be required. Table 5 shows the modulation case table for the two-material multilayered cube using the material mix with the highest compressive strength from the GFC and MC category and the control mix. Figure 4a shows the configuration of the two-layer cube. It has a core encased by an outer layer; both are assigned different material properties and are tie-constrained. The boundary condition is fixed at the base, and a specified displacement is applied to the top surface. Figure 4b(i) displays the axial contour plot of the loaded model. The displacement intensity appears to be higher at the loading face relative to the bottom fixed face. The model’s stress (Misses) contour distribution shown in Figure 4b(ii) indicates a maximum intensity at the top and bottom faces with minimal effects around the mid-section. The stress map validates the compression damage distribution in Figure 4b(iii), which has variation in damage index across the concrete matrix compared to a monolithic cube.

### 3.4. Influence of Layer Volume

The influence of layer volume can be observed in three categories: from Case 1 (MC2-and C) in Figure 4c, Case 2 (GFC-1 and C) in Figure 4d, and Case 3 (GFC-1 and MC-2) in Figure 4e. The thicknesses of the layers are adjusted to correspond to a predetermined volumetric ratio, as outlined in Table 5. The specific dimensions are presented in Table A5 in the Appendix A.

In Figure 4c, C is the outer layer, while MC-2 is the cube’s core. The results show a variation in strength and a strain shift as layer volume changes. Peak strength is achieved at 25% outer volume of C, closely followed by 75% outer volume of C. The minimum strength is at a 50% C outer volume to 50% MC-2 inner volume combination. The results indicate that the performance of a structure using MC-2 as a primary material can be optimized by encasing it with 25% C as no significant improvement in strength is observed by increasing the volume of C as an outer layer. Similarly, in Figure 4d, the outer layer is C, and the inner layer is GFC-1. The variation in strength is evenly spread across the volumetric ratios, with the trend indicating an increase in strength with an increasing outer volume of C.

The overall trend observed in Case 2 differs from Case 1 even though the peak strengths of GFC-1 and MC-2 are not far apart, further indicating that the difference may be owed to the stress–strain behavior of the individual materials, as opposed to the peak strength alone. Both GFC-1 and C individually exhibit a sudden drop in peak strength, fracturing similarly, unlike MC-2, which gently declines in strength. This factor may be responsible for the difference in overall trend between the cases. Figure 4e presents the results of a GFC-1 and MC-2 layering in Case 3. The variation in peak compressive strength is small in magnitude compared to Cases 2 and 1. The stress–strain plot is also equally influenced by the respective material performances, evident by extended strains and multiple peaks. The maximum and minimum compressive strengths observed are at an outer layer of 25% and 75% MC-2, respectively, showing that strength increases with a decrease in MC-2 volume, given that it is positioned as an outer layer.

### 3.5. Influence of Layer Positions and Volume Reversal

To study the influence of layer position, the material volumes from Cases 1 to 3 are reversed. Figure 4f presents the results of Case 4, the polar opposite of Case 1. The results show a glaring trend. The strengths from Case 1 are almost equally reversed. The sudden drop in post-failure strength observed in case one is reduced drastically in Case 4, and the peak strength is now observed in a 50% MC-2 outer volume to 50% C inner volume combination. This shows that the trend of the stress–strain curve is influenced by the outer 50–75% volume/layer while the inner layer or core significantly alters the peak strengths. More so, contrary to Case 1, the minimum strengths are observed in 25% outer volume of MC-2, closely followed by 75% outer volume of MC-2. In Figure 4g, the results of the performance of Case 5 are presented. In the same vein as in Case 2, the variation in strength is evenly spread across the volumetric ratios. The trend indicates an increase in strength with an increasing outer volume of GFC-1. The peak values in compressive strength of the respective combinations in Case 5 are the flip opposite of Case 2 as the volumetric ratios are alternated. Nevertheless, the overall strain at compressive strength remains unchanged. Figure 4f,g reveals that, for a high-to-low strength layer combination, the reversal of layer positions or volumes is tantamount to a mirroring of the initial performance.

### 3.6. Beyond a Two-Layer Configuration

**Figure 5:**
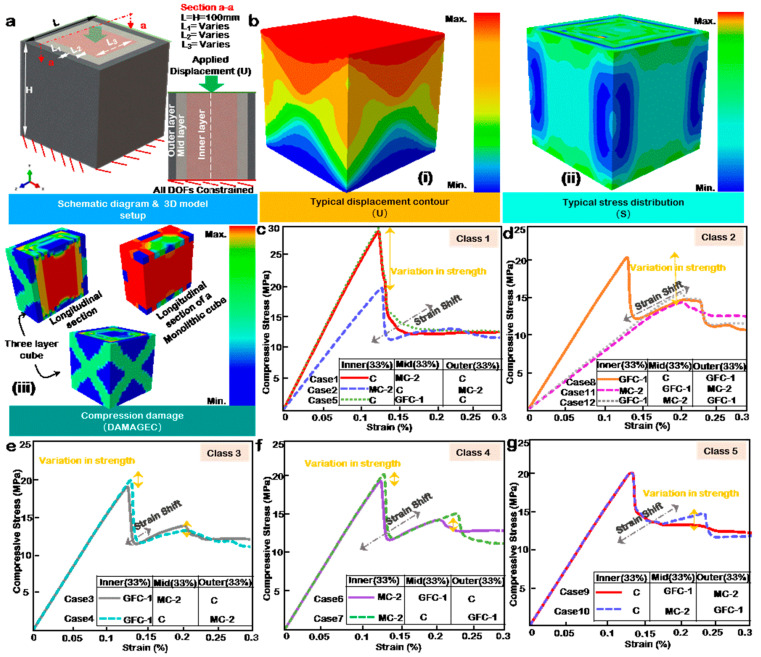
(**a**) Schematic diagram of the three-layer configuration, showing the applied displacement and boundary condition. (**b**) Three-layer configuration simulation contour plot of (**i**) Displacement, (**ii**) Misses stress, and (**iii**) Compression damage. Numerical stress–strain curve for (**c**) Class 1, (**d**) Class 2, (**e**) Class 3, (**f**) Class 4, and (**g**) Class 5.

In the study of layered concrete, it is justified to question what may lie beyond the bi-layer configurations and study the influence of multilayering in three or more layers or finely tuned functionally graded concrete. This study thus further examines a three-layer concrete matrix as presented in Figure 5. Table 6 presents the case table for the study. This case development principle is that the same material properties cannot be allotted to two consecutive layers with direct interaction. Therefore, the largest and most feasible combinations were generated and classified according to their configuration similarities.

The concrete matrix is divided into equal volumes, approximately 33.33% each, as shown in Figure 5a. The model is given the same boundary and loading conditions as in the two-layer and monolithic material cases. Figure 5b(i) shows the displacement plot. In contrast to Figure 4b(i), the distribution differs. In Figure 5b(ii), the typical stress distribution is presented. It can be observed that the edge is isolated from significant stresses, and the stresses are evenly distributed on the faces and the sides, unlike Figure 4b(ii), where the stresses at the sides are also minimal. The damage intensity varies significantly from the two-layer configuration, as shown in Figure 5b(iii). The stress transfer between layers and damage variable for each material differs from the other. This may be the factor responsible for the minimal damage observed in the outer layer of the three-material model.

Figure 5c,d shows the results of three configurations each, categorized as classes 1 and 2. In this category, the governing factor is material volume. Although the materials are split into three separate and equal volumetric layers, one material dominates the volume of the matrix; as a result, its strength and stress–strain behavior influence the overall performance of the model. Thus, for example, in class 1, Cases 1 and 5 show maximum and relatively equal strength and trend because the overall volume in those models is C-dominated. Similarly, classes 2, 11, and 12 present the minimum strength in the class because MC-2 and GFC-1, respectively, dominate them. Figure 5e–g present classes 3–5, respectively, and are similarly configured. However, MC-2 and C alternate positions with GFC-1 as the inner layer in classes 4 and 5, respectively. The resulting strength and stress–strain trends in classes 3–5 are relatively the same, implying that, for a three-layer material model where all layers are from different materials but with the same volumetric composition, the position has minimal influence.

## 4. Conceptual Applications

**Figure 6:**
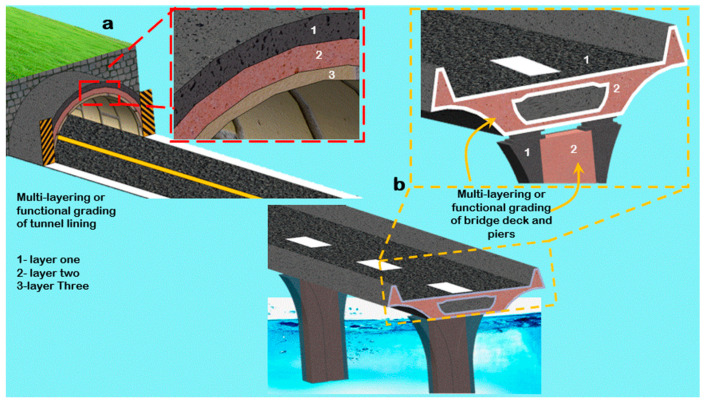
Conceptualization of the application of multilayering to (**a**) tunnels and (**b**) bridges.

Highly compressive structures such as bridges and tunnels can be designed using the PDA with lessons from multilayering. As a result, they can be high-performance-based and cost-efficient. In addition, the nature of arched structures is such that the loads are efficiently transferred, and failure modes are predictable [59]. As such, multilayering can use high-strength fiber concrete, interlaced with ductile high-strength microsilica concrete for structures such as the tunnels and bridges in Figure 6a, minimizing the overall thickness of the structures and maximizing performance. Furthermore, this technique can also be used for the high-fatigue needs of highways by multilayering eco-friendly-rubberized concrete with fiber-concrete, resulting in a more resilient and lightweight structure.

## 5. Conclusions

Although experimental and theoretical frameworks are the foundations of engineering research, numerical tools and FE analysis have played a significant role in civil engineering design, analysis, and prediction in recent years. They help reduce cost and maximize efficiency. Multilayered concrete as a subset of functionally graded concrete, with increasing applicability in 3D printing concrete, also seeks to maximize structural efficacy by creating high performance, fit for purpose, concrete layer configuration. This study has briefly summarized the underlying principles of some existing FE numerical tools and applied them. Experiments on single-material 3D printed cubes using some cementitious composites mixes are conducted. The stress–strain data are converted to numerical input to enable the numerical performance evaluation of multilayered concrete cubes. The FE models are validated against the experimental results. The major conclusions on the material’s performances are as follows:

- The addition of glass fiber to concrete can improve the strain performance as multi-strength peaks and fairly extended strains. However, the addition of glass fiber to 3D printed concrete will influence printability and reduce the mechanical performance of the resulting printed concrete by the unavoidable imperfection of surface and internal voids.
- The addition of microsilica to concrete has the effect of a stretched-out post-failure regime. In other words, it considerably reduces the brittle failure of concrete by sustaining a large strain at maximum load. However, in 3d printing, microsilica can result in a highly cohesive mix, lacking adequate followability, thus yielding a highly stratified sample after printing resulting in print imperfections such as bulking or necking.

On the multilayering front, the layer volume and position influence the performance of multi-layer concrete because stress distribution varies across the concrete matrix. Damage is usually more pronounced at the outer surface than the inner because the inner matrix is confined by the outer. As a result, under compressive loads, the outer surfaces fracture more visibly. When the effects of layer volume and position from a holistic perspective are considered, the following conclusion is drawn:

- For a two-layer configuration, the difference in the respective material strengths greatly influences the role of position and material volume. In other words, when the difference in material strength is high (Case 1, Case 2, Case 4, and Case 5), the stress–strain plot flips with position interchange, as shown in Cases 1 and 4 in Figure 4c,f. However, when the difference in strength is low, the position’s role is minimal, and layer volume governs the performance.
- When the stress–strain performances of the combined materials are similar, the resulting stress–strain plot amplifies the weaknesses and strengths of the source materials.
- The stress–strain behavior of the outer layer of concrete sample influences to a great extent the post-failure performance of the sample. Thus, in multilayering of concrete, the post-failure performance objective should influence the choice of materials regarding positioning. In addition, the peak strength should guide decision-making regarding volumetric ratios.
- A three-layer configuration model is influenced by volume if two of the three layers are allotted the same material. Furthermore, layer position has minimal to zero influence on the concrete models’ performance, provided each layer is allotted a different material and has equal volume.

The application of multilayering in scaled structures is yet to be accessed. In service, some of the challenges of multilayering are stresses–strains transfer between layers, layer interface debonding, and cracking due to stress localization, while in numerical work, modeling interlayer boundary conditions to account for variation in stiffnesses is a challenge. For this study, a simple “tie-constraint” was applied. The stress contribution from layers and the effect of the interlayer boundary condition are presented in the Appendix A. In future studies, multilayering will be applied to scaled structures to assess performance in service. Furthermore, debonding and interlayer stresses will be studied using stress–strain sensors such as piezoelectric wires.

## Figures and Tables

**Table 1:** Microsilica composition.

Constituent		SiO_2_	Fe_2_O_3_	AL_2_O_3_	CaO + MgO	C	K_2_O + Na_2_O
**Weight percentage (%)**		>88	<1.0	<1.0	<0.7	<1.5	<1.5
**Property**	Loss on Ignition	Moisture	PH value (pH)	Specific surface area (m^2^/kg)	Specific gravity		
**Value**	<3%	<2%	4.5–8.5	15	2.3		

**Table 2:** Physical properties of glass fiber.

Material	Length (mm)	Filament Diameter (mm)	Tensile Strength (MPa)	Elastic Modulus (MPa)	Density
Glass fiber	3–6	0.5	1500–1700	72,000	2500

**Table 3:** Mix composition (Kg/m^3^).

Category	Tag	Quarry Dust	OPC	Microsilica	Glass Fiber (% vol.)	SRI
Control (C)	Control	900	600	80	-	0.94
Glass Fiber Concrete (GFC)	GFC-1	835	600	160	1.0	0.98
	GFC-2	835	600	160	1.5	0.96
	GFC-3	835	600	160	2.0	0.94
Microsilica Concrete (MC)	16%MC-1	846	600	144	0.5	0.97
	23%MC-2	792	600	207	0.5	0.96
	30%MC-3	739	600	270	0.5	0.96

**Table 4:** Comparison of the Abaqus CSC, CDP, and the Diana FEA MFC models.

	Model Type	Post-Failure Stiffness	Yield Function	Flow Rule	Crack Detection	Damage Recovery (Loading and Unloading)
**CSC** [50]	Continuum, smeared cracking. Cracks are fixed and limited to one per integration point	Damaged-elasticity, strain hardening, and tension softening	Simple compression yield surface & the coulomb line	Associated: over predicts inelastic volume strain	Orientation storage of individual cracks, then “plastic “strains of cracks are calculated	Cracks can close completely when stress across them becomes compressive. Not designed for cyclic loading
**MFC** [51]	Continuum, smeared cracking. It can be rotating or fixed with more than three cracks and up to a maximum of six cracks per integration point	MCC curves and a selection of tension stiffening or softening curves	Reduces the shear modulus of the concrete by K until the failure limit is reached	Governed by plastic dilatancy, a function of the first strain invariant and K	Makes use of K. Stores the orientation of individual cracks; then, stresses are evaluated in the direction of the respective cracks	The crack reclosing option can be defined
**CDP** [52]	Continuum plasticity-based. Fracture energy cracking criterion can be initiated for tensile failure in unreinforced/under-reinforced concrete.	Damaged-elasticity, strain hardening and tension stiffening or softening	Drucker–Prager criteria	Non-associated. Hyperbolic flow potential: fairly accurate	No direct detection. Softening begins beyond tensile failure stress	Compressive stiffness is recoverable upon cracks closure as the loads transition from tensile to compressive. Designed for cyclic loading

**Table 5:** Two-material multi-layer concrete case table.

	Inner (Layer Volume: 25%,50%,75%)			
**Outer** **(layer volume: 25%,50%,75%)**		**Control (C)**	**MC-2**	**GFC-1**
	**Control**	C	Case 1: C_MC-2	Case 2: C_GFC-1
	**MC-2**	Case 4: MC-2_C	MC-2	Case 3: MC-2_GFC-1
	**GFC-1**	Case 5: GFC-1_C	Case 6: GFC-1_MC-2	GFC-1

**Table 6:** Three-material multi-layer concrete case table.

	Inner (Layer Volume: 33.333% Each)			
**Outer and mid** **(layer volume: 33.333% each)**		**Control (C)**	**MC-2**	**GFC-1**
	**Control_MC-2**	Class 1–Case 1: C_MC-2_C	Class 1–Case 2: MC-2_C_MC-2	Class 3–Case 3,4: C_MC-2_GFC-1 MC-2_C_GFC-1
	**Control_GFC-1**	Class 1–Case 5: C_GFC-1_C	Class 4–Case 6,7: C-GFC-1_MC-2 GFC-1_C_MC-2	Class 2–Case 8: GFC-1_C_GFC-1
	**MC-2_GFC-1**	Class 5–Case 9,10: MC-2_GFC-1_C GFC-1_MC-2_C	Class 2–Case 11: MC-2_GFC-1_MC-2	Class 2–Case 12: GFC-1_MC-2_GFC-1

**Figure A1:**
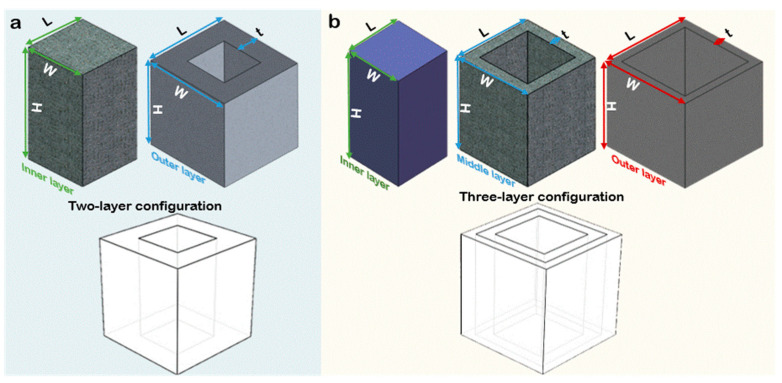
Configuration and parameters of (**a**) two-layer model and (**b**) three-layer model.

**Figure A2:**
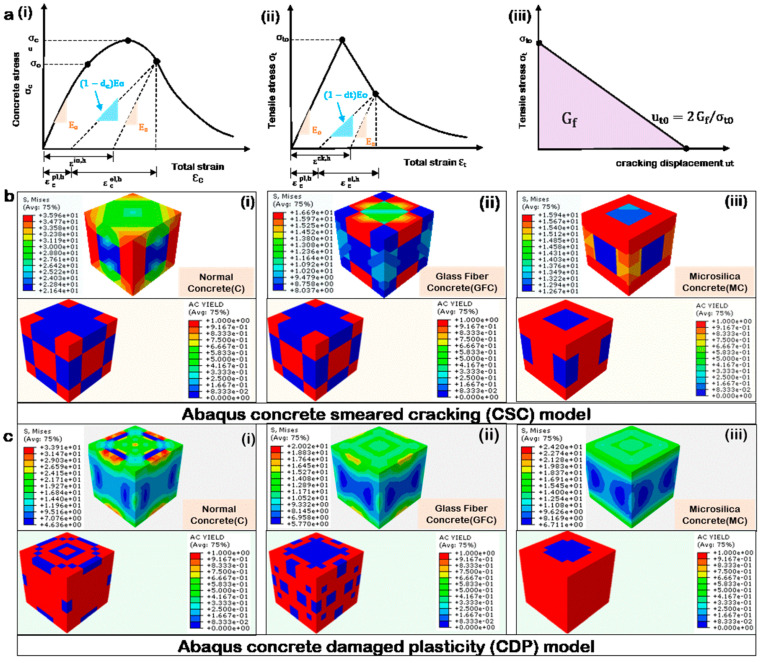
(**a**) The typical response of concrete to uniaxial loading in (**i**) Compression, (**ii**) Tension, and (**iii**) Fracture energy cracking criterion. (**b**) Failure stress and yield results for CSC model of (**i**) C, (**ii**) GFC-1, and (**iii**) MC-2. (**c**) Failure stress and yield results for CDP model of (**i**) C, (**ii**) GFC-1, and (**iii**) MC-2.

**Figure A3:**
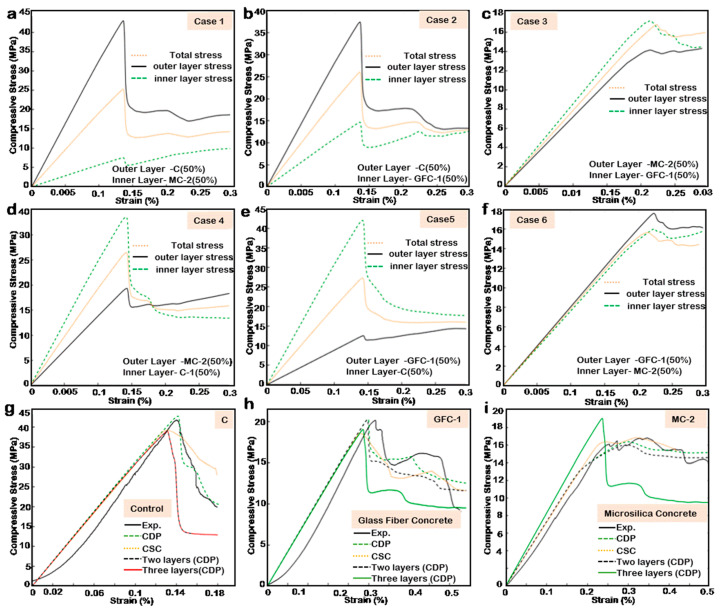
Numerical stress–strain curve of a two-layer configuration showing stress contribution from the inner and outer layers in (**a**) Case 1, (**b**) Case 2, (**c**) Case 3, (**d**) Case 4, (**e**) Case 5, and (**f**) Case 6. Effect of the “tie-constraint” interlayer boundary condition on (**g**) Control sample, (**h**) GFC-1, and (**i**) MC-2.

## Data Availability

Data are available from the corresponding author on reasonable request.

## Appendix A

The dimensions and configurations of the multi-layer cubes are presented in Figure A1. Table A1 presents the values of the parameters.

**Table A1:** Dimensions of the multi-layer model.

Configuration	Volume (%)	Position	Width W (mm)	Length L (mm)	Height H (mm)	Thickness t (mm)
Two-layer	25	Inner	50.00	50.00	100.00	-
	75	Outer	100.00	100.00	100.00	25
	50	Inner	70.72	70.72	100.00	-
	50	Outer	100.00	100.00	100.00	14.64
	75	Inner	86.60	86.60	100.00	-
	25	Outer	100.00	100.00	100.00	6.7
Three-layer	33	Inner	57.73	57.73	100.00	-
	33	Middle	81.65	81.65	100.00	11.95
	33	Outer	100.00	100.00	100.00	9.175

### Appendix A.1. Compressive Stress–Strain Modeling

The compressive stress strength for uniaxial loading can be classified into two regimes: the plastic and elastic regimes; the total strain tensor $\varepsilon_{c}$ is comprised of the elastic part $\varepsilon^{\mathrm{el}}$ and the plastic part $\varepsilon^{\mathrm{pl}}$ as shown in Figure A2a(i). For modeling in ABAQUS, only yield stresses ($\sigma_{c}$)—inelastic strains ($\varepsilon^{\mathrm{in},h}$) or plastic stains ($\varepsilon^{\mathrm{pl},h}$) are required. A corresponding damage parameter data set is relevant for CDP models, while tension stiffening data is required for CSC models. The first value of the yield stresses must correspond to a zero inelastic/plastic strain. Therefore, total compressive strain values $\left(\varepsilon_{c}\right)$ from experiments should be converted to inelastic or plastic strains as required.

(A1) $$\varepsilon^{\mathrm{in},h}=\varepsilon_{c}-\varepsilon_{\mathrm{oc}}^{\mathrm{el}}$$

where $\varepsilon_{\mathrm{oc}}^{\mathrm{el}}=\frac{\sigma_{c}}{E_{o}}$. $\varepsilon_{\mathrm{oc}}^{\mathrm{el}}$ is the elastic strain corresponding to the undamaged concrete, and $E_{o}$ is the modulus of elasticity.

In CDP models, damage parameter (d_c_) needs to be generated corresponding to the yield stresses at the various inelastic strains defined as

(A2) $$d_{c}=1-\frac{\sigma_{c}}{\sigma_{\mathrm{cu}}}$$

where $\frac{\sigma }{\sigma_{c}}$ is the fraction of remaining stress to stress at cracking and $\varepsilon -\varepsilon_{c}$ is the absolute value of the direct strain minus the direct strain at cracking. $\sigma_{\mathrm{cu}}$ is the peak yield stress. Care should be taken to ensure that the plastic strain values $\varepsilon^{\mathrm{pl},h}$ determined using Equation (2) are neither negative nor decreasing with increased stresses [60,61]. In the same way as damage parameters were defined for CDP modeling, tension stiffening parameters need to be provided for CSC models as $\frac{\sigma }{\sigma_{c}}$ corresponding to $\varepsilon -\varepsilon_{c}$.

### Appendix A.2. Tensile Stress–Strain Modeling

As discussed in the compressive stress–strain model, the concrete tensile damage plasticity model as shown in Figure A2a(ii) for an experimentally tested concrete material can be derived from the stress–total strain data as follows. The cracking strain should be calculated from the total strain defined as

(A3) $$\varepsilon^{\mathrm{ck},h}=\varepsilon_{t}-\varepsilon_{\mathrm{ot}}^{\mathrm{el}}$$

where $\varepsilon_{\mathrm{ot}}^{\mathrm{el}}=\frac{\sigma_{t}}{E_{o}}$. $\varepsilon_{\mathrm{ot}}^{\mathrm{el}}$ is the elastic strain corresponding to the undamaged concrete and $\varepsilon_{t}$ is the total tensile strain, after which a damage parameter $(d_{t}$) is generated corresponding to the yield stresses at the various cracking strains as [60,61]

(A4) $$d_{t}=1-\frac{\sigma_{t}}{\sigma_{\mathrm{to}}}$$

Alternatively, where a tensile test is not performed, with the fracture energy (G_F_) and cracking displacement $\left(u_{t}^{\mathrm{ck}}\right)$, complete loss of strength of the material takes place. See Figure A2a(iii). It can be defined analytically corresponding to the yield tensile strength $\left(\sigma_{\mathrm{to}}\right)$ using Equations (7)–(9).

(A5) $$\sigma_{\mathrm{to}}=0.3016\;{\left(f_{\mathrm{ck}}\right)}^{\frac{2}{3}}$$

(A6) $$G_{F}=0.073f_{\mathrm{cm}}{}^{0.18}$$

(A7) $$u_{t}^{\mathrm{ck}}=2G_{f}/\sigma_{\mathrm{to}}$$

### Appendix A.3. Boundary Conditions, Solvers, Loading Rate and Input Data

The numerical model was set up in geometric and boundary similitude to the experimental testing program in Abaqus CAE. The base of the cube was fully constrained. All displacements (U1, U3) and rotation (UR1, UR2, UR3) of the loaded face were set to zero except the displacement in the axial direction (U2), which was set to a specified displacement. The Static General solver and Static Riks step were used in the case of CDP and CSC models, respectively. The loading rate was consistent with the experimental program. A coarse mesh seeding of 25 was used for the CSC model to enable adequate convergence beyond the elastic regime. The CDP model was minimally sensitive to mesh size. The parameters for simulating the compression behavior of C, GFC-1, and MC-2 are presented in Table A2, Table A3 and Table A4, respectively. Table A5 presents the data for concrete damage plasticity, density, young’s modulus, and poisson’s ratio. For multi-layer concrete, the layers were tie constrained to force equal deformation and stress transfer at the layer interface. The stress distribution contour of the model using the CSC and CDP model is compared in Figure A2b,c, respectively. Good agreements are obtained.

**Table A2:** Concrete compression and tension damage data for the control sample.

Compression				Tension			
Yield Stress (N/mm^2^)	Inelastic Strain	Damage Parameters	Direct Strain	Yield Stress (N/mm^2^)	Fracture Energy (N.mm/mm^2^)	Damage Parameters	Displacement Rate
27.4663 #	0 #	0	0.00095	3.531510255	0.1418483	0	0
40.0668 *	6.949 × 10^−5^ *	0	0.0014 *			0.99	1
33.1797	0.00044819	0.1718	0.00155				
23.4759	0.00084542	0.4140	0.001625				
20.8940	0.00105616	0.4785	0.00175				
17.2352	0.00142766	0.5698	0.002				
10.1460	0.00196307	0.7467	0.0023				

# Input starting point for CDP model. * Direct strain at cracking/input starting point for CSC model.

**Table A3:** Concrete compression and tension damage data for the GFC-1.

Compression				Tension			
Yield Stress (N/mm^2^)	Inelastic Strain	Damage Parameters	Direct Strain	Yield Stress (N/mm^2^)	Fracture Energy (N.mm/mm^2^)	Damage Parameters	Displacement Rate
15 #	0 #	0	0.0019	3.028683453	0.124446573	0	0
19.36424 *	7.947 × 10^−5^ *	0	0.0025 *			0.99	1
11.07944	0.0016150	0.427840	0.003				
11.49108	0.00206361	0.406582	0.0035				
11.95366	0.00250579	0.382694	0.004				
7.5821	0.00355223	0.608448	0.0045				

# Input starting point for CDP model. * Direct strain at cracking/input starting point for CSC model.

**Table A4:** Concrete compression and tension damage data for the MC-2.

Compression				Tension			
Yield Stress (N/mm^2^)	Inelastic Strain	Damage Parameters	Direct Strain	Yield Stress (N/mm^2^)	Fracture Energy (N.mm/mm^2^)	Damage Parameters	Displacement Rate
12.4455 #	0 #	0	0.0019	1.7672209	0.117664076	0	0
16.47673 *	4.618 × 10^−5^ *	0	0.0024*			0.99	1
14.96941	0.0007615	0.0914817	0.0029				
15.84147	0.0011369	0.0385549	0.0034				
14.18350	0.0018737	0.1391801	0.0039				
12.32112	0.002639	0.2522110	0.0044				
12.18422	0.0031593	0.2605198	0.0049				
11.529645	0.0037529	0.3002470	0.0054				

# Input starting point for CDP model. * Direct strain at cracking/input starting point for CSC model.

**Table A5:** Concrete damage plasticity, density, young’s modulus, and poisson’s ratio.

Dilation Angle (°)	Eccentricity	fB0/fc0	K	Viscosity Parameter	Density (t/mm^3^)	Young’s Modulus (N/mm^2^)			Poisson’s Ratio
**35**	**0.1**	1.16	0.67	0.007985	1.81 × 10^−9^	30,000 (C)	8000 (GFC-1)	7000 (MC-2)	0.22

### Appendix A.4. Stress Contributions and Effect of Interlayer Boundary Condition

The stress contribution from the inner layer and outer layer of a two-layer configuration is presented for Cases 1 to 6 in Figure A3a–f. It can be observed that the total stress is an average of stresses from the layers. Therefore, the stress performance of a multi-layer compression structure is partly dependent on the stress performance of individual layers. Figure A3g–i presents the effect of the “tie-constraint” boundary condition on the performance of multi-layer model for the control, GFC-1 and MC-2 materials using the CDP model. The elastic regime of the models appears to be consistent irrespective of the number of layers; however, the non-linear phase of the stress–strain curve deviates from the initial monolithic model in the multi-layer model. For the control material, the deviation from the monolithic sample is constant for both the two-layer model and the three-layer model as shown in Figure A3g. For GFC-1 and MC-2, there is more deviation in the three-layer model relative to the two-layer model, as shown in Figure A3h,i. Thus, for a multi-layer model in compression, the inelastic performance is dependent on the interlayer boundary condition, and the overall performance is a factor of the individual layers and interlayer bond/boundary conditions.
